# Role of N6-methyladenosine methylation in nasopharyngeal carcinoma: current insights and future prospective

**DOI:** 10.1038/s41420-024-02266-y

**Published:** 2024-12-18

**Authors:** YaYan Zhao, Jie Li, MeiJuan Dian, YaNan Bie, ZhiTao Peng, Ying Zhou, BingQian Zhou, WeiChao Hao, XiCheng Wang

**Affiliations:** 1 Department of Oncology, The First Affiliated Hospital of Guangdong Pharmaceutical University, Guangzhou, China; 2 Cancer Research Institute of Integrated Traditional Chinese and Western Medicine, The First Affiliated Hospital of Guangdong Pharmaceutical University, Guangzhou, China; 3 Department of Thoracic Surgery, Nanfang Hospital, Southern Medical University, Guangzhou, China; 4 School of Basic Medical Sciences, Guangdong Pharmaceutical University, Guangzhou, China; 5 Cancer Research Institute, School of Basic Medical Sciences, Southern Medical University, Guangzhou, China

**Keywords:** Cancer epigenetics, Head and neck cancer

## Abstract

Nasopharyngeal carcinoma (NPC) is a distinct type of head and neck squamous cell carcinoma prevalent in Southern China, Southeast Asia, and North Africa. Despite advances in treatment options, the prognosis for advanced NPC remains poor, underscoring the urgent need to explore its underlying mechanisms and develop novel therapeutic strategies. Epigenetic alterations have been shown to play a key role in NPC progression. Recent studies indicate that dysregulation of RNA modifications in NPC specifically affects tumor-related transcripts, influencing various oncogenic processes. This review provides a comprehensive overview of altered RNA modifications and their regulators in NPC, with a focus on m^6^A and its regulatory mechanisms. We discuss how m^6^A RNA modification influences gene expression and affects NPC initiation and progression at the molecular level, analyzing its impact on cancer-related biological functions. Understanding these modifications could reveal new biomarkers and therapeutic targets for NPC, offering promising directions for future research and precision medicine.

## Facts

- Dysregulated RNA modification regulators shape tumor-specific epitranscriptomic landscapes in NPC.
- m^6^A modifications regulate both post-transcriptional and transcriptional processes, interacting with histone modifications and chromatin states, closely linked to NPC progression.
- RNA modifications, particularly m^6^A, play critical roles in diverse biological processes essential to NPC progression.

## Open Questions

- How can RNA modification patterns serve as biomarkers for predicting NPC outcomes and therapeutic responses?
- What roles do less-explored RNA modifications (e.g., m^1^A, Nm, ac^4^C) and their regulators play in NPC progression?
- What mechanisms govern the crosstalk between RNA modifications and other epigenetic changes, such as DNA methylation, histone modifications, and chromatin remodeling?
- How do RNA modifications modulate intercellular communication within the tumor microenvironment?
- How do EBV-driven epitranscriptomic changes contribute to NPC initiation and progression?

## Introduction

Nasopharyngeal carcinoma (NPC) is a distinctive type of head and neck squamous cell carcinoma (HNSCC) originating from the epithelial mucosa of the nasopharynx. It is particularly prevalent in Southern China, Southeast Asia, and North Africa, with age-standardized incidence ranging from 4 to 25 cases per 100,000 individuals, as reported by GLOBOCAN [1, 2]. In recent decades, certain endemic regions have experienced a decline in NPC incidence and mortality, with average annual reductions of approximately 1% to 5%, largely attributed to changes in lifestyle and environmental factors [3, 4]. However, treatment options remain limited. The primary first-line therapy is intensity-modulated radiotherapy (IMRT) combined with concurrent or neoadjuvant chemotherapy [5–7], while anti-PD-1 immunotherapy has emerged as the most effective novel option for advanced-stage disease [8–12]. Moreover, the prognosis for advanced-stage NPC remains poor, emphasizing the need for novel therapeutic strategies.

Epstein-Barr virus (EBV) infection is a major etiological factor in NPC pathogenesis, while HLA gene variants, located in the coding region of MHC class I molecules on chromosome 6p21, represent key genetic contributors [13, 14]. Decades of research have also recognized epigenetic dysregulation as a critical driver in cancer development. Epigenetic alterations, including DNA methylation and histone modifications, are heavily implicated in NPC progression [15]. Among these, aberrant DNA methylation is particularly prevalent in NPC, with hypermethylation frequently observed in the promoter CpG islands of tumor suppressor genes (TSGs) [15]. These methylation changes can be detected in tissue, brushing, and blood samples from NPC patients [16–20]. EBV contributes to this epigenetic reprogramming through its oncogenic proteins, such as latent membrane protein 1 (LMP1), which induces DNA methylation by upregulating DNA methyltransferases (DNMTs) 1, 3a, and 3b [21–23]. Methylation of TSG promoters impacts various cellular functions and pathways, including cell cycle regulation and proliferation (e.g., RASSF1A [24], CDH4 [25], MIPOL1 [26], ARNTL [27], PTPRG [28], ZNF671 [29], miR-31 [30], and miR-34c [31]), invasion and metastasis (e.g., RASSF2A [32], DNAJA4 [33], HOPX [34], NEURL3 [35], ZNF582 [36], RAB37 [37], and SHISA3 [38]), apoptosis and necroptosis (e.g., UCHL1 [39], CMTM3 [40], and RIP3 [22]), radioresistance (e.g., USP44 [41]), ERK/NF-κB signaling (e.g., RERG [42]), and Wnt/β-catenin signaling (e.g., SOX1 [43]). Although histone modification in NPC is less extensively studied, it remains crucial. Elevated H3K27me3 levels in NPC tissues compared to normal nasopharyngeal epithelium (NPE) are associated with poor patient prognosis and contribute to chemoresistance and radioresistance [44, 45]. This elevation is driven by EZH2, a key component of the polycomb repressive complex 2 (PRC2) with histone-lysine N-methyltransferase activity, which promotes NPC cell survival, metastasis, and angiogenesis [15, 46, 47]. EBV also influences histone methylation by creating a bivalent switch with increased H3K27me3 and decreased H3K4me3, contributing to the repression of DNA damage repair genes [48]. The H3K9me3 demethylase KDM4A is overexpressed in NPC, correlating with poor survival by reducing H3K9me3 enrichment at the HIF1α promoter, thus promoting HIF1α expression and NPC progression [49]. Histone deacetylase 4 (HDAC4) is similarly upregulated in NPC, where it binds to the E-cadherin promoter, repressing its transcription and driving tumor progression and metastasis [50]. EBV LMP1 upregulates STAT5A and recruits HDAC1/2 to the CEBPA locus, reducing histone acetylation and repressing CEBPA transcription, leading to a dedifferentiated, stem-like state [51].

In addition to the aforementioned, epitranscriptomic regulation adds another level of epigenetic control over gene expression. Over 170 types of RNA chemical modifications have been identified [52]. Within recent decades, advances in transcriptome-wide sequencing techniques have enabled detailed insights into the RNA modification landscape [53]. Numerous studies on RNA modification modifiers—writers, erasers, and readers—have shown that these RNA modifications are dynamic and reversible, and play a critical role in RNA metabolism, influencing a wide range of physiological and pathological processes. In cancer, alterations in RNA modifications and their regulatory machinery have been observed, exerting either oncogenic or tumor-suppressive effects [52]. In this review, we present an overview of the RNA modification landscape, with a focus on m^6^A and its altered modifiers in NPC. Furthermore, we summarize the current understanding of how RNA modification regulators influence molecular outcomes and mechanisms in NPC. Finally, we discuss the role of RNA modifications in cancer-related biological functions and their significance in NPC.

## Aberrant RNA modification and clinical implications in NPC

### Landscape of RNA modifications in NPC

Recent studies have brought RNA modification to the forefront of epigenetic research, revealing its extensive involvement in cancer. Unlike DNA and histone modification, RNA modification can occur both co-transcriptionally and post-transcriptionally, adding a layer of complexity to gene regulation [54]. The frequency of RNA modifications often varies significantly between tumors and normal tissues or cells, correlating with different patient outcomes or disease stages [55]. Among these modifications, N6-methyladenosine (m^6^A) is the most well-studied modification, present in various RNA subspecies such as mRNAs, lncRNAs, miRNAs, their precursors, and rRNAs [56]. In addition to m^6^A, other chemical modifications such as N1-methyladenosine (m^1^A), 5-methylcytosine (m^5^C), 5-hydroxymethylcytidine (hm^5^C), N7-methylguanosine (m^7^G), N6,2’-O-dimethyladenosine (m^6^Am), 2’-O-methylated nucleotides (Nm), N4-acetylcytosine (ac^4^C), and pseudouridine (Ψ) are also found in different RNA types. These modifications influence critical molecular processes, including translation, splicing, RNA stability, and nuclear export, affecting both normal physiology and pathological processes like cancer [53].

In mRNAs, m^6^A modifications are commonly found around the stop codon, within the coding region, and the 5′ untranslated region (UTR). In NPC, recent findings reveal specific patterns of m^6^A modifications. In NPE, the incidence of m^6^A modifications is 10.62% in the 5’ UTR, 63.07% in the CDS, and 26.31% in the 3’ UTR. In NPC, the distribution of m^6^A modifications shows little variation: in primary NPC, 7.44% are in the 5’ UTR, 62.52% in the CDS, and 30.04% in the 3’ UTR; in recurrent NPC, 9.65% are in the 5’ UTR, 63.22% in the CDS, and 27.14% in the 3’ UTR [57]. Despite the similar distribution patterns, the overall levels of m^6^A modifications are significantly higher in NPC compared to NPE [58, 59]. In primary and recurrent NPC, 1465 and 1613 transcripts, respectively, show significantly higher levels of m^6^A modification compared to NPE. The expression levels of many of these transcripts also vary, indicating m^6^A-mediated regulation [57].

Non-coding RNAs (ncRNAs), which do not encode proteins, play crucial roles in regulating cellular processes. They include miRNAs, lncRNAs, circular RNAs (circRNAs), small nuclear RNAs (snRNAs), and ribosomal RNAs (rRNAs). RNA modifications are key regulators of ncRNA function. For example, m^6^A modifications can influence the processing and splicing of miRNA precursors, essential for the generation of mature miRNAs [60]. In lncRNAs, m^6^A can alter regulatory functions through the “m^6^A-switch” mechanism, which modifies RNA-protein interactions by changing the local structure of lncRNAs. Significant differences in m^6^A modifications are observed between NPC and NPE in ncRNAs. For example, in primary NPC, 1167 lncRNAs, 176 pre-miRNAs, 134 pri-miRNAs, and 99 small nucleolar RNAs (snoRNAs) show altered m^6^A modification levels compared to NPE. In recurrent NPC, 534 lncRNAs, 57 pre-miRNAs, and 63 pri-miRNAs have different m^6^A modifications compared to primary NPC [57].

Ribosomal RNA (rRNA) accounts for over 80% of total RNA, making it a major component of cellular RNA. Thus, m^6^A modifications in rRNA’s large and small subunits comprise a significant portion of RNA modifications. Emerging evidence suggests that these epigenetic modifications are crucial for ribosome biogenesis, which occurs in the nucleolus where rRNAs and proteins assemble into ribosomes for mRNA translation. Aberrant ribosome synthesis is often seen in tumorigenesis and cancer progression, contributing to uncontrolled cell growth. Specifically, in NPC, the m^6^A modification at position A1832 of 18S rRNA is notably increased in cell lines and tissue samples compared to normal controls [61]. These observations indicate that m^6^A modifications in rRNA might be vital for the onset and progression of NPC, similar to their roles in other RNA types.

In addition to m^6^A, other RNA modifications, such as m^5^C, m^7^G and ac^4^C, have distinct effects based on the RNA type and specific modification. Although studies have documented diverse RNA modifications in various cancers, research in NPC remains sparse. Notably, global m^7^G modifications in tRNA are significantly higher in NPC tissues and cell lines, correlating with poor prognosis in advanced cases [62], suggesting its role in the severity of the disease. There is, however, a lack of direct evidence regarding other RNA modifications in NPC, highlighting the need for further investigation in this area.

### Writers

**Fig. 1:**
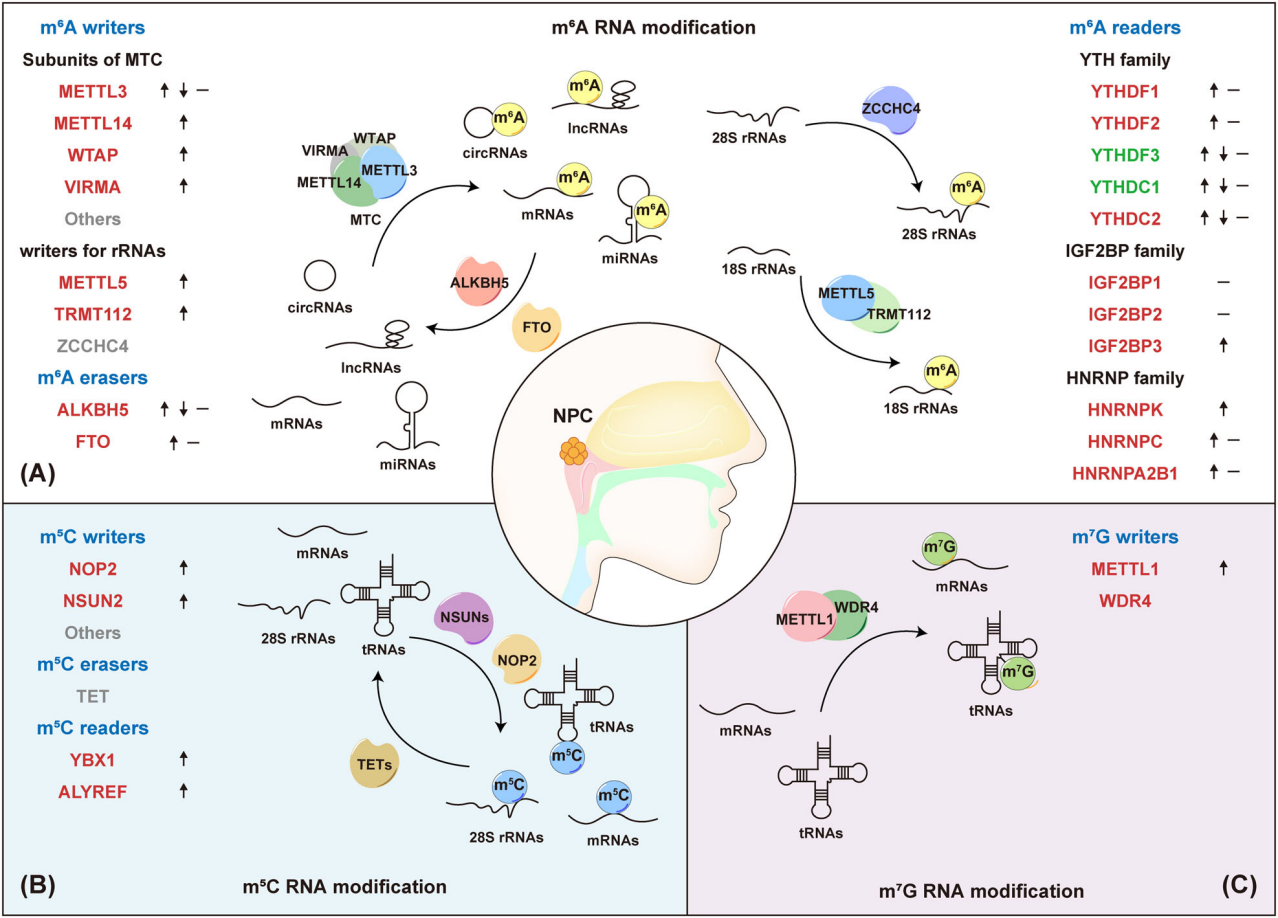
Expression and oncogenic roles of RNA modification regulators in NPC. **A m**^**6**^**A RNA modification:** The methyltransferase complex (MTC), composed of METTL3, METTL14, WTAP, VIRMA, and additional subunits, primarily catalyzes m^6^A modifications on RNA. Two demethylases, FTO and ALKBH5, remove m^6^A marks from modified RNAs. Additional m^6^A methyltransferases, such as the METTL5-TRIM112 complex and ZCCHC4, specifically add m^6^A modifications to 18S and 28S rRNAs, respectively. Reader proteins recognize m^6^A sites, including YTH domain-containing proteins (YTHDF1/2/3, YTHDC1/2), IGF2BP1/2/3, and HNRNPs. **B m**^**5**^**C RNA modification:** m^5^C methylation is catalyzed by RNA-type-specific NSUN family proteins and NOP2, while TET family enzymes remove m^5^C marks. Reader proteins YBX1 and ALYREF recognize m^5^C modifications. **C m**^**7**^**G RNA modification:** The METTL1-WDR4 complex installs m^7^G modifications on mRNAs and tRNAs. Oncogenic proteins in NPC are highlighted in red, tumor suppressors in green, and proteins with undefined roles in gray. Upward arrows indicate proteins upregulated in NPC, while downward arrows denote downregulated proteins.

Indeed, chemical modifications of RNAs are tightly controlled by a complex machinery that includes writers, erasers, and readers. Disruption of this dynamic equilibrium leads to aberrant gene expression and function, thereby contributing to cancer development. Deposition of m^6^A on mRNA, lncRNA and miRNA is catalyzed by the METTL3-METTL14 methyltransferase complex, where METTL3 acts as the main catalytic subunit and METTL14, WTAP and VIRMA serve as the RNA-binding scaffold that recognizes the substrate (Fig. 1A).

Consistent with the observed alterations in m^6^A modifications across various mRNAs, lncRNAs, and miRNAs, research shows that m^6^A writer proteins are significantly altered in NPC. Studies on METTL3 expression in NPC are inconsistent: some report increased levels [59, 63–66], while others find decreased levels [67] or no change [58] (Fig. 1A). EBV infection can downregulate METTL3 [68, 69]. Despite these discrepancies, most studies agree that METTL3 contributes to NPC malignancies [59, 64–66, 70, 71] (Fig. 1A and Table 1). In contrast to METTL3, METTL14 [57, 58, 63, 72], WTAP [57, 58, 63, 73], and VIRMA [63, 74, 75] are consistently upregulated in NPC compared to normal NPE tissues, and their high expression levels are associated with poor prognosis (Fig. 1A).

**Table 1:** Roles of m^6^A regulators in NPC.

Regulator	Role	Targets	Regulatory Effect		Downstream Signaling Pathway	Function	Ref.
**m**^**6**^**A writers**							
**MTC subunits**							
**METTL3**	Oncogene	Snail	mRNA stability ↑	-		EMT, migration, invasion and metastasis	[64]
		TNKS	mRNA stability ↑	AXIN/β-catenin/TCF signaling		migration, invasion and metastasis	[65]
		ZNF750	mRNA decay ↑	FGF14		proliferation and apoptosis	[130]
		TRIM11	mRNA stability ↑	Daple/DVL/β-catenin/ABCC9		chemoresistance and p62-selective autophagy	[180]
		pri-miR-19a	pri-miRNA splicing ↑	DGCR8/BAMBI		proliferation and migration	[59]
		miR-1908-5p	miRNA stability ↑	HOPX		proliferation and migration	[70]
		ZFAS1	lncRNA stability ↑	miR-100-3p/ATG10/PI3K-AKT signaling		proliferation, autophagy and EMT	[63]
		LINC00313	lncRNA stability ↑	PTBP1/STIM1/AKT-mTOR signaling		cancer stemness and autophagy	[66]
		FAM225A	lncRNA stability ↑	miR-590-3p, miR-1275/ITGB3/FAK-PI3K-AKT signaling		proliferation, migration, invasion and metastasis	[145]
		SUCLG2‐AS1	lncRNA stability ↑	CTCF/SOX2		proliferation, migration, invasion, metastasis and radioresistance	[71]
**METTL14**	Oncogene	ANKRD22	mRNA stability and translation ↑	SLC25A1/Acetyl-CoA		lipid metabolism, proliferation, migration, invasion and metastasis	[58]
		AOC1	mRNA stability ↑	-		proliferation and migration	[72]
**WTAP**	Oncogene	TEAD4	mRNA stability ↑	BZW2/PHLPP2/AKT signaling		migration, invasion, metastasis and chemoresistance	[103]
		DIAPH1-AS1	lncRNA stability ↑	MTDH/LASP1		proliferation, migration, invasion and metastasis	[111]
**VIRMA**	Oncogene	E2F7	mRNA stability ↑	CBFB-RUNX1/ITGA2, ITGA5, NTRK1/PI3K-AKT signaling		proliferation, migration, invasion and metastasis	[74]
		PTGS2	mRNA stability ↑	-		proliferation, migration and invasion	[75]
		LINC00839	lncRNA stability ↑	TAF15/AOC1		proliferation, migration, invasion and metastasis	[110]
**rRNA writers**							
**METTL5/TRMT112 complex**	Oncogene	18S rRNA	Translation ↑	HSF4b/HSP90B1/mutant p53		proliferation, migration, invasion and chemoresistance	[61]
**m**^**6**^**A erasers**							
**ALKBH5**	Oncogene	ARHGAP35	mRNA stability and translation ↓	-		proliferation, migration and metastasis	[80]
**FTO**	Oncogene	ARHGAP35	mRNA stability and translation ↓	-		proliferation, migration and metastasis	[80]
		CD44 precursors	pre-mRNA splicing ↓	CD44V		ferroptosis and radioresistance	[82]
		OTUB1	mRNA stability ↑	SLC7A11		ferroptosis and radioresistance	[81]
**m**^**6**^**A readers**							
**YTH family**							
**YTHDF2**	Oncogene	TEAD4	mRNA stability ↑	BZW2/PHLPP2/AKT signaling		migration, invasion, metastasis and chemoresistance	[103]
**YTHDF3**	Repressor	CBX1	mRNA decay ↑	H3K9me3/MAP7 IFN-γ/STAT1/PD-L1		proliferation, migration, invasion, metastasis and immune evasion	[84]
**YTHDC1**	Repressor	CD44 precursors	pre-mRNA splicing ↑	CD44V		ferroptosis and radioresistance	[82]
**YTHDC2**	Oncogene	IGF1R	mRNA translation ↑	AKT signaling		radioresistance	[86]
**IGF2BP family**							
**IGF2BP1**	Oncogene	AKT2	mRNA stability ↑	-		taxol-resistance	[109]
	Oncogene	LINC00839	lncRNA stability ↑	TAF15/AOC1		proliferation, migration, invasion and metastasis	[110]
	Oncogene	LINC00313	lncRNA stability ↑	PTBP1/STIM1/AKT-mTOR signaling		cancer stemness and autophagy	[66]
**IGF2BP2**	Oncogene	Snail	mRNA stability ↑	-		EMT, migration, invasion and metastasis	[64]
		TRIM11	mRNA stability ↑	Daple/DVL/β-catenin/ABCC9		chemoresistance and p62-selective autophagy	[180]
		E2F7	mRNA stability ↑	CBFB-RUNX1/ITGA2, ITGA5, NTRK1/PI3K-AKT signaling		proliferation, migration, invasion and metastasis	[74]
		ANKRD22	mRNA stability and translation ↑	SLC25A1/Acetyl-CoA		lipid metabolism, proliferation, migration, invasion and metastasis	[58]
		DIAPH1-AS1	lncRNA stability ↑	MTDH/LASP1		proliferation, migration, invasion and metastasis	[111]
**IGF2BP3**	Oncogene	KPNA2	mRNA stability ↑	EMT		proliferation, migration, invasion and metastasis	[87]
		NOTCH3	mRNA stability ↑	Notch signaling		metastasis and cancer stemness	[85]
		SUCLG2‐AS1	lncRNA stability ↑	CTCF/SOX2		proliferation, migration, invasion, metastasis and radioresistance	[71]
		ZNF750	mRNA decay ↑	FGF14		proliferation and apoptosis	[130]
**HNRNP family (roles in NPC mediated by m**^**6**^**A remain unverified)**							
**HNRNPK**	Oncogene	MMP12	Transcrption ↑	-		migration and invasion	[93]
		FLIP	Transcrption ↑	-		apoptosis	[94]
		TP	mRNA stability ↑	-		apoptosis	[92]
**HNRNPA2B1**	Oncogene	-	-	-		proliferation, migration and invasion	[97]
**HNRNPC**	Oncogene	circITCH	-	miR-224-3p		proliferation and migration	[96]

The m^6^A modification of 18S and 28S rRNA is mediated by the METTL5-TRMT112 complex and ZCCHC4, respectively. METTL5, with TRMT112, specifically methylates adenine at position 1832 of 18S rRNA [76] (Fig. 1A). In NPC, this modification is significantly increased compared to normal controls. Both METTL5 and TRMT112 show elevated expression in NPC, particularly in tumors with distant metastasis, correlating with advanced disease stages [61, 77] (Fig. 1A). The status of m^6^A modification in 28S rRNA or the expression of its writer, ZCCHC4 in NPC remains unknown, which requires further investigation (Fig. 1A).

m^5^C is an important RNA modification affecting various RNA types, regulated by specific writers (NOP2, NSUN2-7, TRDMT1), erasers (TETs), and readers (YBX1, ALYREF) based on RNA type and modification sites (Fig. 1B). Analysis of GEO RNA-seq datasets (GSE12452, GSE53819, and GSE61218) shows that NOP2, NSUN2, YBX1, and ALYREF levels are generally higher in NPC than in normal tissues [78, 79]. An immunohistochemistry (IHC) study corroborates the increased expression of NSUN2 and ALYREF in NPC samples. Notably, high levels of NOP2 and NSUN2, and ALYREF are associated with poor prognosis. NSUN2 and ALYREF serve as independent risk factors for both overall survival and disease-free survival, and are linked to tumor staging and distant metastasis [78, 79] (Fig. 1B). Additionally, transcriptome analysis indicates that NSUN2 might reduce immune cell infiltration in the tumor microenvironment and contribute to chemotherapy resistance [78].

m^7^G is a prevalent modification at the mRNA cap and in internal mRNA, microRNA, tRNA, and rRNA. Within tRNA, m^7^G46 is the most frequently methylated site, located in the variable loop region. Here, m^7^G46 stabilizes the tRNA structure by forming a tertiary base pair with C13-G22. The METTL1/WDR4 complex primarily mediates this modification (Fig. 1C). The role of m^7^G in cancer has become increasingly recognized, as this modification is highly expressed across various cancers, including lung cancer, hepatocellular carcinoma, and bladder cancer. In NPC, METTL1 levels are notably elevated compared to normal tissues. Patients with post-treatment distant metastasis exhibit significantly higher METTL1 expression than those without metastasis. METTL1 expression correlates with disease progression, increasing from early stages to recurrent or metastatic disease. Higher METTL1 levels are associated with advanced disease stages and are linked to poorer outcomes, including overall survival, disease-free survival, distant metastasis-free survival, and locoregional relapse-free survival [62] (Fig. 1C).

### Erasers

The m^6^A modification can be removed by two RNA demethylases, FTO and ALKBH5, which act as erasers of this modification (Fig. 1A). Some studies suggest that FTO primarily targets m^6^Am. In NPC tissues, GEO database analyses have shown that ALKBH5 is typically downregulated compared to normal NPE [58, 63, 77], while FTO expression remains largely unchanged [58, 63] (Fig. 1A). However, a recent study observed a trend of hypomethylation in NPC mRNA compared to normal tissues. Immunohistochemistry (IHC) analysis revealed upregulation of ALKBH5 and FTO, which correlated with advanced disease stages and poorer prognosis [80] (Fig. 1A). Notably, Huang et al. found that FTO is upregulated in radioresistant NPC tissues, where it mediates radiotherapy resistance through OTUB1-dependent ferroptosis inhibition [81] (Table 1). Recent findings from the same group revealed that, in radioresistant NPC, lncRNA HOTAIRM1 enhances ferroptosis resistance by regulating FTO acetylation and stability, promoting the alternative splicing of CD44 precursor mRNA [82] (Table 1). This post-translational regulation of FTO may explain the differing trends in eraser expression observed across studies. These variations may also reflect the distinct pathological contexts in NPC, such as radioresistance. Thus, although current research on m^6^A erasers in NPC is limited, these findings present promising directions for future studies.

### Readers

The functions and mechanisms of m^6^A writers and erasers are primarily mediated by various m^6^A readers, including members of the YTH domain-containing family, the insulin-like growth factor 2 mRNA-binding protein (IGF2BP) family, and heterogeneous nuclear ribonucleoprotein (HNRNP) family. The YTH domain-containing family were the first m^6^A readers identified, each with specific molecular functions. For example, YTHDF2 facilitates mRNA degradation, YTHDF1 enhances mRNA translation, and YTHDF3 can regulate both mRNA decay and translation [83]. Current research lacks sufficient evidence to suggest specific expression changes in YTH family proteins between NPC and NPE, or among different stages of NPC. Studies so far have been limited to gene expression analysis in public RNA-seq datasets, often showing inconsistent trends. For example, in six GEO datasets (GSE64634, GSE12452, GSE13597, GSE34573, GSE53819, and GSE68799), YTHDF1 is upregulated only in GSE34573, YTHDF2 is upregulated in three datasets (GSE12452, GSE34573, and GSE53819), and YTHDF3 is upregulated in four datasets (GSE12452, GSE34573, GSE53819, and GSE68799) [67, 77] (Fig. 1A). However, YTHDF3 is also reported to be downregulated in NPC in different studies [84, 85], and positively correlated with patient prognosis in datasets GSE68799 and GSE102349 [67, 84] (Fig. 1A). Additionally, YTHDC1 shows both upregulation and downregulation in GSE34573 and GSE53819 respectively, while YTHDC2 is downregulated only in GSE68799 [67, 77] (Fig. 1A). The TCGA database shows that YTHDF1-3 and YTHDC1 are elevated in NPC compared to NPE, while YTHDC2 shows no significant changes [63] (Fig. 1A). In other hand, a separate study finds that YTHDC2 is elevated in patient specimens with higher radioresistance, correlating with more advanced clinical stages and worse prognosis [86] (Fig. 1A).

IGF2BP1, IGF2BP2, and IGF2BP3, another group of m^6^A readers, recognize m^6^A-modified RNA via their KH domains. These proteins are highly expressed in various cancer types and participate in numerous molecular mechanisms. In most public datasets, IGF2BP1 and IGF2BP2 do not show significant increases in NPC compared to NPE [67, 77] (Fig. 1A). However, IGF2BP3 expression is significantly higher in NPC tissues than in normal tissues, particularly in metastatic NPC [67, 77, 85, 87, 88] (Fig. 1A). High IGF2BP3 levels are associated with poor overall survival (OS) and distant metastasis-free survival (DMFS) in NPC patients [85, 87, 88] (Fig. 1A).

In addition to direct recognition by RNA-binding proteins (RBPs), m^6^A has an ability of “m^6^A-switch”, altering local RNA structure to either facilitate or impede the binding of certain RBPs. The HNRNP family consists of RNA-binding proteins that been named hnRNPA1-U on the basis of their molecular weight. Several studies on HNRNP proteins, including HNRNPC, HNRNPG and HNRNPA2B1, underscore the crucial role of the “m^6^A-switch” mechanism in their RNA splicing regulation [89–91]. Multiple findings suggest involvement of HNRNP proteins in NPC. For example, cytoplasmic HNRNPK exhibits a high expression in NPC compared to NPE and correlating with progression and poor prognosis of NPC [92] (Fig. 1A). It functions as an upstream modulator of diverse oncogenes, including TP, FLIP2, and MMP2 [92–95] (Table 1). In public GEO datasets, HNRNPC is upregulated in three out of six datasets (GSE12452, GSE13597, and GSE34573), and HNRNPA2B1 in two out of six datasets (GSE12452 and GSE34573) [67, 77] (Fig. 1A). Research have reported HNRNPC interacts with and reduces circITCH, which sponges miR-224-3p, thereby contributing to NPC tumorigenesis [96] (Table 1). HNRNPA2B1 is also a driver of NPC, serving as a functional target of miR-146b-5p and SOX2-OT [97] (Fig. 1A and Table 1). However, the current studies do not extensively elucidate the involvement of m^6^A in these processes, necessitating further clarification.

## Mechanisms and molecular effects of m^6^A RNA modifications in NPC

### Regulation of RNA stability and decay

**Fig. 2:**
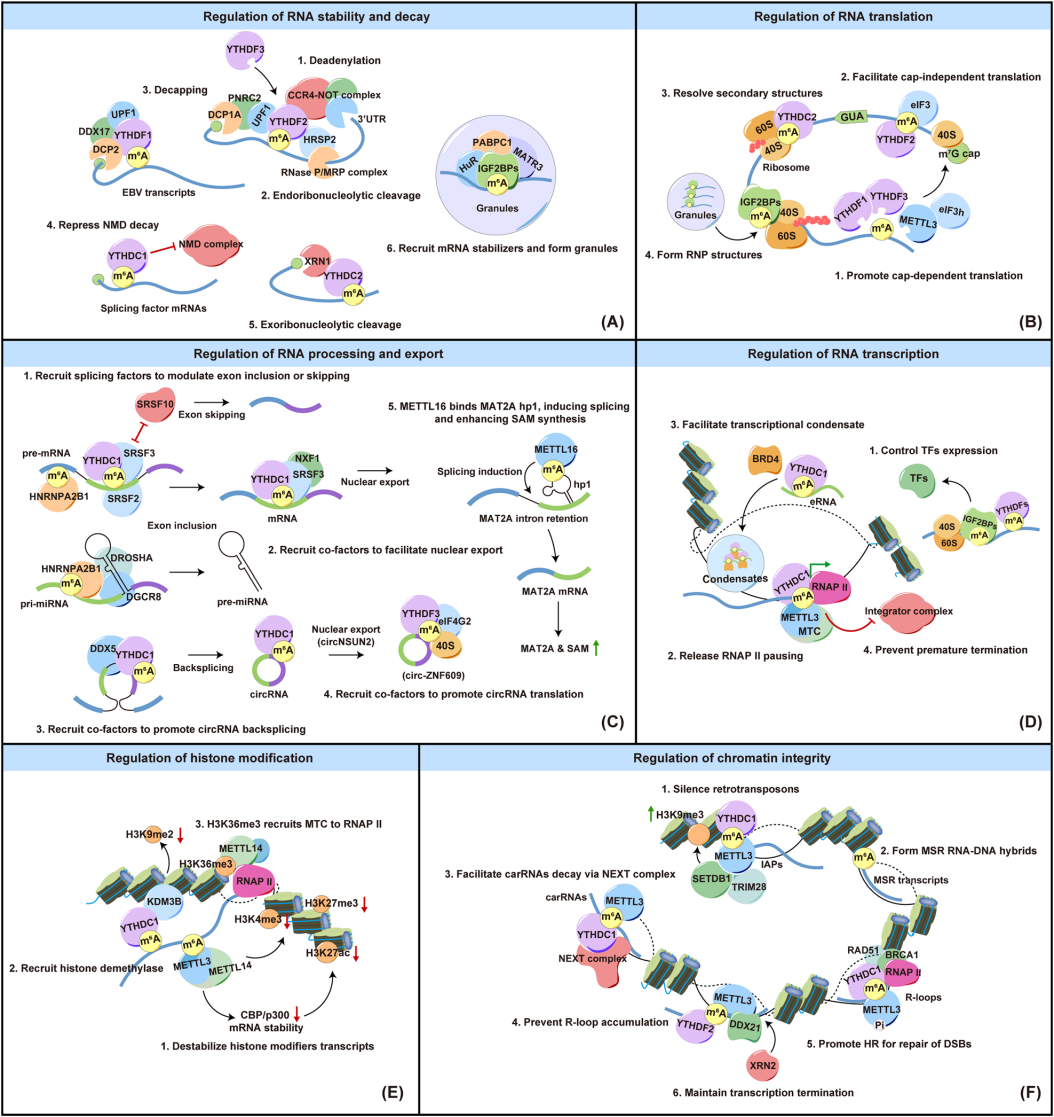
Roles of m^6^A modifications in gene regulation. **A RNA stability and decay**. (1) YTHDF2 promotes decay of m^6^A-modified RNA by recruiting: (1) the CCR4-NOT complex for deadenylation, (2) RNaseP/MRP endonucleases for cleavage, and (3) UPF1/PNRC2/DCP1A for decapping. YTHDF1 facilitates EBV transcripts degradation. YTHDF3 interacts with YTHDF2 to support mRNA decay. (4) YTHDC1 stabilizes splicing factor mRNAs by inhibiting NMD via m^6^A. (5) YTHDC2 promotes mRNA decay through XRN1-mediated exoribonucleolytic cleavage. (6) The IGF2BP family stabilizes mRNAs by recruiting HuR, MATR3, and PABPC1, forming protective ribonucleoprotein granules under stress. **B RNA translation**. (1) YTHDF1 promotes cap-dependent translation by connecting m^6^A-modified mRNAs to ribosomes, coordinated by YTHDF3. METTL3 supports translation initiation via eIF3h, facilitating mRNA circularization. (2) eIF3 also enables cap-independent translation by targeting m^6^A in the 5′-UTR. Under stress, YTHDF2 maintains 5′ UTR methylation for selective mRNA translation. (3) YTHDC2 aids translation elongation by resolving m^6^A-modified structures. (4) IGF2BPs enhance translation by forming ribonucleoprotein granules. **C RNA processing and export**. (1) YTHDC1 regulates splicing by recruiting SRSF2/3 for exon inclusion or SRSF10 for exon skipping, with HNRNPA2B1 also influencing splicing. HNRNPA2B1/m^6^A aids miRNA formation by directing the DGCR8-DROSHA complex to pri-miRNAs. (2) YTHDC1 facilitates the export of m^6^A-modified mRNAs to the cytoplasm through SRSF3 and NXF1, while also directing the export of circRNAs, such as circNSUN2. (3) YTHDC1 recruits DDX5 to assist in the backsplicing required for circRNA formation. (4) YTHDF3 and eIF4G2 regulate circ-ZNF609 translation. (5) METTL16 supports SAM levels by promoting splicing of retained introns in MAT2A. **D Transcriptional regulation**. (1) m^6^A modifications are critical for regulating transcription by controlling TF expression. (2) m^6^A influences RNAP II pausing at promoters by recruiting MTC and YTHDC1, facilitating RNAP II release. (3) m^6^A marks on eRNAs attract YTHDC1 to form condensates crucial for BRD4 activation. (4) MTC-deposited m^6^A modifications at promoters and enhancers prevent early integrator-mediated termination, ensuring robust transcription. **E Histone modifications**. (1) METTL14-mediated m^6^A destabilizes transcripts for histone modifiers, influencing histone marks such as H3K27me3, H3K27ac, and H3K4me3 in neural stem cells. (2) YTHDC1 recruits KDM3B to demethylate H3K9me2, promoting gene expression. (3) H3K36me3, mediated by SETD2, attracts METTL14 to facilitate global m^6^A deposition on actively transcribed RNAs. **F Chromatin integrity**. (1) METTL3 and YTHDC1, along with SETDB1 and TRIM28, uphold heterochromatin integrity by silencing retrotransposons. (2) m^6^A stabilizes chromatin by promoting MSR transcript association. (3) YTHDC1-mediated destabilization of methylated carRNAs regulates local chromatin states. (4) In R-loops, m^6^A recognized by YTHDF2 prevents R-loop accumulation, which can lead to genomic instability and R-loop-dependent DSBs. (5) At DSB sites, ATM-mediated phosphorylation of METTL3 recruits YTHDC1 to safeguard RNA-DNA hybrids, facilitating RAD51, BRCA1, and RNAP II-mediated homologous recombination repair. (6) DDX21 anchors METTL3 for m^6^A deposition at transcription termination sites, recruiting XRN2 to ensure transcription termination and maintain genomic stability.

RNA modification critically influences RNA stability and degradation, though the effects are often complex. Early studies revealed that m^6^A-modified RNAs are generally less stable than their unmodified counterparts, reducing their abundance [98, 99]. Subsequent research established that YTHDF2 actively promotes m^6^A-marked RNA decay by recruiting the CCR4-NOT complex [100] (Fig. 2A). YTHDF2 also interacts with HRSP12 to recruit RNaseP/MRP endoribonucleases for transcript cleavage [101] (Fig. 2A). Additionally, YTHDF2 facilitates PNRC2-mediated decapping via UPF1 [102] (Fig. 2A). However, in NPC, YTHDF2 binding to m^6^A-modified TEAD4 stabilizes the transcript, driving NPC development [103] (Table 1), demonstrating the context-dependent nature of YTHDF2 regulation. YTHDF1, while typically enhancing the translation of m^6^A-modified mRNAs, can also recruit RNA degradation complexes such as ZAP, DDX17, and DCP2 in EBV-infected cells, to accelerate the degradation of viral transcripts [104] (Fig. 2A and Table 2). YTHDF3 promotes mRNA decay through interactions with YTHDF2 [83] (Fig. 2A). In NPC, YTHDF3 destabilizes m^6^A-modified CBX1 mRNA, acting as a tumor suppressor [84] (Table 1). Outside of YTHDF proteins, m^6^A modifications at the start codon stabilize splicing factor mRNAs by inhibiting nonsense-mediated mRNA decay (NMD) in glioblastoma, mediated by YTHDC1 [105] (Fig. 2A). YTHDC1 also drives the decay of m^6^A-marked chromosome-associated regulatory RNAs (carRNAs) via the nuclear exosome targeting (NEXT) complex [106]. Additionally, YTHDC2 regulates mRNA stability through its interaction with the exoribonuclease XRN1 [107] (Fig. 2A). IGF2BP family proteins (IGF2BP1, IGF2BP2, IGF2BP3) stabilize mRNAs in an m^6^A-dependent manner by recruiting stabilizing proteins like HuR, MATR3, and PABPC1. They may also form cytoplasmic RNP granules to protect mRNAs under stress [108] (Fig. 2A). In NPC, IGF2BP1 stabilizes m^6^A-modified transcripts like AKT2, LINC00839, and LINC00313 [66, 109, 110], while IGF2BP2 stabilizes E2F7 and DIAPH1-AS1 [74, 111] (Table 1). IGF2BP3 protects KPNA3 and NOTCH3 from degradation [87, 88] (Table 1).

**Table 2:** Crosstalk between m^6^A regulators and EBV in cancer.

Regulator	Tumor Type	Functional Role	Targets	Regulatory Effect on RNA	Function	EBV-Mediated Regulation	Ref.
**m**^**6**^**A writers**							
**METTL3**	NPC	Suppressor	KLF4	RNA decay ↑	Infection and replication	EBV/BZLF1 decreases METTL3	[69, 104]
	B lymphoma	Supporter	EBNA2	-	Infection	-	[196]
		Supporter	BZLF1, BHRF1, BMRF1, BKRF4, BALF4	RNA stability (BZLF1, BHRF1, BMRF1) and RNA translation (BKRF4, BALF4) ↑	Lytic replication	-	[198]
		Suppressor	TLR9	RNA translation ↑	Tumor immune	EBV/EBNA1 decreases METTL3	[68]
	EBVaGC	Supporter	SNAIL1, ZMYM1, SOCS2	-	Tumor proliferation, migration and invasion	EBV-circRPMS1/Sam68 increases METTL3	[204]
**METTL14**	NPC	Suppressor	-	-	Infection and replication	-	[104]
	B lymphoma	Supporter	EBNA3C	RNA stability ↑	EBV-related gene regulation and tumor proliferation	Latent infection/EBNA3C increases METTL14, lytic reactivation decreases METTL14	[199]
**WTAP**	EBVaGC	Suppressor	-	-	Tumor proliferation and migration	EBV/EBER1 decreases WTAP	[202]
**m**^**6**^**A erasers**							
**ALKBH5**	NPC	Supporter	-	-	Infection and replication	-	[104]
	B lymphoma	Suppressor	TYK2, DTX4	RNA decay (TYK2) and RNA translation (DTX4) ↓	Lytic replication and IFN response suppression	Lytic reactivation decreases ALKBH5	[197, 199]
**FTO**	B lymphoma	Suppressor	EBNA2	-	Infection	-	[196]
	EBVaGC	Suppressor	FOS	RNA stability ↓	Tumor migration and invasion	EBV increases FTO	[203]
**m**^**6**^**A readers**							
**YTHDF1**	NPC	Suppressor	BZLF1, BRLF1	RNA decay ↑	Infection and replication	EBV decreases YTHDF1	[104]
	B lymphoma	Supporter	EBNA2	-	Infection	-	[196]
		Suppressor	TLR9	RNA translation ↑	Tumor immune	EBV/EBNA1 decreases METTL3	[68]
**YTHDF2**	NPC	Suppressor	KLF4	RNA decay ↑	Infection and replication	-	[69, 104]
	B lymphoma	Suppressor	EBNA2	-	Infection	-	[196]
		Suppressor	ZTA, RTA, BGLF4, CASP8	RNA decay ↑	Lytic replication	Lytic reactivation decreases YTHDF2	[200]
		Suppressor	ZTA, RTA, BALF5, BGLF4, BLLF1	RNA decay ↑	Replication	-	[201]
		Supporter	TYK2	RNA decay ↑	Lytic replication and IFN response suppression	Lytic reactivation decreases YTHDF2	[197, 199]
**YTHDF3**	NPC	Suppressor	-	-	Lytic replication	-	[104]
		Supporter	IFITM1	RNA decay ↑	Infection	-	[195]
	B lymphoma	Suppressor	EBNA2	-	Infection	-	[196]
**IGF2BP1/2**	EBVaGC	Supporter	FOS	RNA stability ↑	Tumor migration and invasion	-	[203]

### Regulation of RNA translation

While mRNAs are directly transcribed from genes, proteins are the ultimate output of gene expression. m^6^A modification influences mRNA translation into proteins through various mechanisms. YTHDF1 enhances cap-dependent translation by recruiting eukaryotic initiation factor 3 (eIF3) and linking m^6^A-modified mRNAs to ribosomes [112] (Fig. 2B). YTHDF3 further supports this by interacting with ribosomal subunits and coordinating with YTHDF1 [113] (Fig. 2B). METTL3, as m^6^A reader, promotes translation initiation by interacting with eIF3h, enabling mRNA circularization [114] (Fig. 2B). eIF3 also facilitates cap-independent translation by directly binding to m^6^A in the 5′-UTR of transcripts [115] (Fig. 2B). YTHDC2 facilitates translation elongation by resolving secondary structures within coding sequences via m^6^A modifications [116] (Fig. 2B). IGF2BPs stabilize or enhance the translation of target RNAs, often through granule-like ribonucleoprotein structures in the perinuclear region [108, 117] (Fig. 2B). Under stress conditions, YTHDF2 helps regulate selective mRNA translation by preserving 5′ UTR methylation, which promotes cap-independent translation during heat shock [118] (Fig. 2B). In NPC, FTO and ALKBH5 demethylate m^6^A-modified ARHGAP35, reducing its translation and contributing to NPC progression [80] (Table 1). YTHDC2 also binds to m^6^A-modified IGF1R mRNA, facilitating translation initiation and contributing to radioresistance [86] (Table 1). The METTL5/TRMT112 complex facilitates m^6^A modification on 18S rRNA at position 1832, aiding the interaction between RPL24 protein and 18S rRNA, thus enhancing 80S ribosome assembly and the translation of mRNAs with 5’ TOP motifs [61] (Table 1).

### Regulation of RNA processing and export

RNA modification plays a crucial role in regulating RNA processing and export. In mRNA, m^6^A accumulates in exons near intronic 5′/3′ splice sites of pre-mRNAs, which flank spliced exons [119] (Fig. 2C). The m^6^A reader YTHDC1 promotes alternative splicing by recruiting and modulating splicing factors like SRSF3 and SRSF10, thereby aiding their access to target mRNA binding sites and encouraging exon inclusion [120] (Fig. 2C). YTHDC1 also facilitates the export of m^6^A-modified mRNA from the nucleus to the cytoplasm by interacting with SRSF3 and NXF1 [121] (Fig. 2C). Likewise, HNRNPA2B1 binds to nuclear transcripts, influencing alternative splicing [90] (Fig. 2C). METTL16 regulates intracellular SAM levels by promoting the splicing of a retained intron in MAT2A mRNA, which encodes the enzyme for SAM synthesis, especially during methionine depletion [122] (Fig. 2C). In NPC, HOTAIRM1 interacts with the FTO protein to induce m^6^A demethylation of the CD44 transcript. Without m^6^A, CD44 is not recognized by YTHDC1, leading to a shift from CD44S to CD44V. Elevated CD44V levels inhibit irradiation-induced ferroptosis and contribute to NPC radioresistance [82] (Table 1).

miRNA maturation occurs in three primary stages: First, RNA polymerase II transcribes miRNA into a long, capped precursor known as primary miRNA (pri-miRNA) within the nucleus. Next, Drosha ribonuclease III and DGCR8 convert pri-miRNA into pre-miRNA. Finally, the pre-miRNA is exported to the cytoplasm, where the RNase III enzyme Dicer cleaves it into mature miRNA [123] (Fig. 2C). Research shows that m^6^A modifications and their regulatory factors are essential in miRNA processing and maturation. During miRNA biogenesis, the m^6^A mark added by METTL3 guides the microprocessor complex, comprising DGCR8 and DROSHA, to recognize and process pri-miRNAs [60] (Fig. 2C). Additionally, HNRNPA2B1 binds to m^6^A marks on specific pri-miRNA transcripts, interacts with DGCR8 within the microprocessor complex, and enhances pri-miRNA processing [90] (Fig. 2C). In NPC, METTL3-mediated m^6^A modifications enhance the processing and maturation of pri-miR-19a through DGCR8, leading to the upregulation of miR-19a-3p. This upregulation suppresses BAMBI expression, thereby promoting NPC cell proliferation and invasion [59] (Table 1).

RNA modifications are also critical in circRNA biosynthesis, export, and translation. For example, METTL3 and YTHDC1 direct the back-splicing necessary for circRNA formation [124, 125] (Fig. 2C). In colorectal cancer, YTHDC1 promotes the export of circNSUN2 to the cytoplasm [126] (Fig. 2C). Additionally, YTHDF3 and eIF4G2 regulate the translation of circ-ZNF609 [127, 128] (Fig. 2C). In NPC, HNRNPC interacts and impairs circITCH expression, facilitating NPC progression by reducing miR-224-3p levels [96] (Table 1).

### Regulation of RNA transcription

Transcriptional regulation is critical in tumorigenesis and progression, influencing cellular state and cancer cell plasticity. Normally, lineage-specific transcription factors (TFs) maintain cellular identity during development. In cancer, however, these TFs often become dysregulated, leading to aberrant activities that disrupt normal cell states and promote malignancy [129]. In NPC, RNA modifications play a crucial role in transcriptional regulation by controlling TF expression (Fig. 2D). For example, the EMT-TF Snail, which drives aggressive cancer traits, is stabilized by m^6^A modification mediated by METTL3 and IGF2BP2 [64] (Table 1). E2F7, stabilized by VIRMA-mediated m^6^A and IGF2BP3, activates ITGA2, ITGA5, and NTRK1 transcriptionally in cooperation with RUNX1 and CBFB, thus facilitating NPC progression [74] (Table 1). TEAD4, stabilized by WTAP-mediated m^6^A and YTHDF2, activates BZW2 transcription, driving NPC malignancy [103] (Table 1). Conversely, ZNF750, a tumor suppressor in NPC, is destabilized by METTL3-mediated m^6^A modification, leading to reduced FGF14 expression and diminished apoptosis-promoting effects [130] (Table 1). Additionally, the METTL5-TRIM112 complex enhances HSF4b translation through 18S rRNA m^6^A modification, activating HSP90B1 transcription. This process involves binding to oncogenic mutant p53, preventing its degradation and promoting NPC tumorigenesis and chemoresistance [61] (Table 1).

Strictly speaking, RNA modification affecting transcription factors is not a direct form of transcriptional regulation, as it operates not during transcription. The transcription process involves three highly regulated stages: initiation, elongation, and termination. m^6^A RNA modification plays a key role in these stages by affecting RNAP II pausing at gene promoters. It does this by recruiting the m^6^A methyltransferase complex (MTC) and YTHDC1, which facilitates the release of RNAP II from its pause and promotes subsequent transcription. This effect relies on the catalytic activity of METTL3 [131] (Fig. 2D). m^6^A modification is also added to nascent enhancer RNAs (eRNAs), where it recruits YTHDC1 to form liquid-like condensates. These m^6^A-eRNA/YTHDC1 condensates are crucial for forming BRD4 coactivator condensates, necessary for enhancer and gene activation [132] (Fig. 2D). Moreover, the MTC-deposited m^6^A modifications on nascent transcripts at promoters and enhancers protect them from premature termination by the integrator complex, thereby promoting productive transcription [133] (Fig. 2D). In NPC, the super-enhancer lncRNA SUCLG2-AS1, stabilized by METTL3-mediated m^6^A modification and IGF2BP3, enhances CTCF occupancy at the SOX2 promoter and enhancers, boosting SOX2 transcription through the formation of long-range chromatin loops, thereby promoting NPC metastasis and increasing radiosensitivity [71] (Table 1).

### Regulation of histone modification

Recent evidence has highlighted the close relationship between m^6^A modification and histone modification. In mouse embryonic neural stem cells, METTL14-mediated m^6^A modification regulates histone marks such as H3K27me3, H3K27ac, and H3K4me3 by destabilizing transcripts encoding histone modifiers, thus affecting genes related to proliferation and differentiation [134] (Fig. 2E). Another study finds that YTHDC1 recruits KDM3B to m^6^A-associated chromatin regions, promoting H3K9me2 demethylation and enhancing gene expression [135] (Fig. 2E). In NPC, CBX1 functions as a reader protein recognizing H3K9me3 histone methylation, which contributes to heterochromatin formation and MAP7 transcriptional inhibition. m^6^A modification of CBX1 aids in its recognition and degradation by YTHDF3. Consequently, decreased YTHDF3 levels in NPC lead to increased CBX1 expression, promoting tumor progression [84] (Table 1). ANKRD22, catalyzed by METTL14 and recognized by IGF2BP2, shows elevated expression and boosts intracellular acetyl-CoA levels (Table 1). This increase further enhances histone acetylation levels, particularly H3K27ac around the METTL14 promoter, creating a positive feedback loop perpetuating malignant progression in NPC [58]. Conversely, histone modification also influences m^6^A RNA modification. SETD2-mediated H3K36me3 recruits METTL14, facilitating the binding of the m^6^A MTC to adjacent RNA polymerase II during transcription elongation. This recruitment ensures m^6^A deposition on actively transcribed nascent RNAs, enhancing m^6^A modification globally [136] (Fig. 2E). In NPC, KAT3A-mediated H3K27ac activates WTAP transcription, causing its upregulation [111] (Table 1). This evidence further implicate interplay between RNA modification and histone modification.

### Regulation of chromatin integrity

The m^6^A modification is crucial for the formation and maintenance of heterochromatin, which is vital for regulating gene expression and maintaining genome integrity [137]. In mouse embryonic stem cells, m^6^A methylation is essential for preserving heterochromatin integrity, particularly at retrotransposons like intracisternal A particle (IAP) elements. METTL3, together with the m^6^A reader YTHDC1 and the H3K9 methyltransferase SETDB1, ensures the proper localization of heterochromatin marks at IAPs, silencing retroviral elements and supporting normal development [138, 139] (Fig. 2F). m^6^A modification also stabilizes heterochromatin by enhancing the chromatin association of MSR transcripts and their ability to form RNA-DNA hybrids, thus maintaining genome integrity and proper gene expression [140] (Fig. 2F). Furthermore, carRNAs can be methylated by METTL3 and destabilized by YTHDC1 through the NEXT complex, fine-tuning nearby chromatin states and downstream transcription [106] (Fig. 2F).

R-loops, structures formed by RNA-DNA hybrids and unpaired single-stranded DNA, are a significant source of genomic instability in mammalian cells. m^6^A modification on RNA within R-loops, regulated by YTHDF2, is crucial for preventing R-loop accumulation, thus safeguarding genomic stability and suppressing R-loop-dependent DNA double-strand breaks (DSBs) [141] (Fig. 2F). When DSBs occur, METTL3, activated by ATM-mediated phosphorylation, localizes to damage sites and methylates RNA to m^6^A. This modification recruits YTHDC1, which protects RNA-DNA hybrids and facilitates the recruitment of RAD51, BRCA1, and RNAP II for homologous recombination (HR)-mediated repair [142] (Fig. 2F). Additionally, DDX21 anchors to R-loops and recruits METTL3 to nascent transcripts, aiding m^6^A deposition at transcription end sites (TESs) and facilitating XRN2 loading for proper transcription termination, thereby maintaining genome stability [143] (Fig. 2F).

## Mechanisms and biological effects of RNA modifications in NPC

### Regulation of oncogenic signaling pathways

**Fig. 3:**
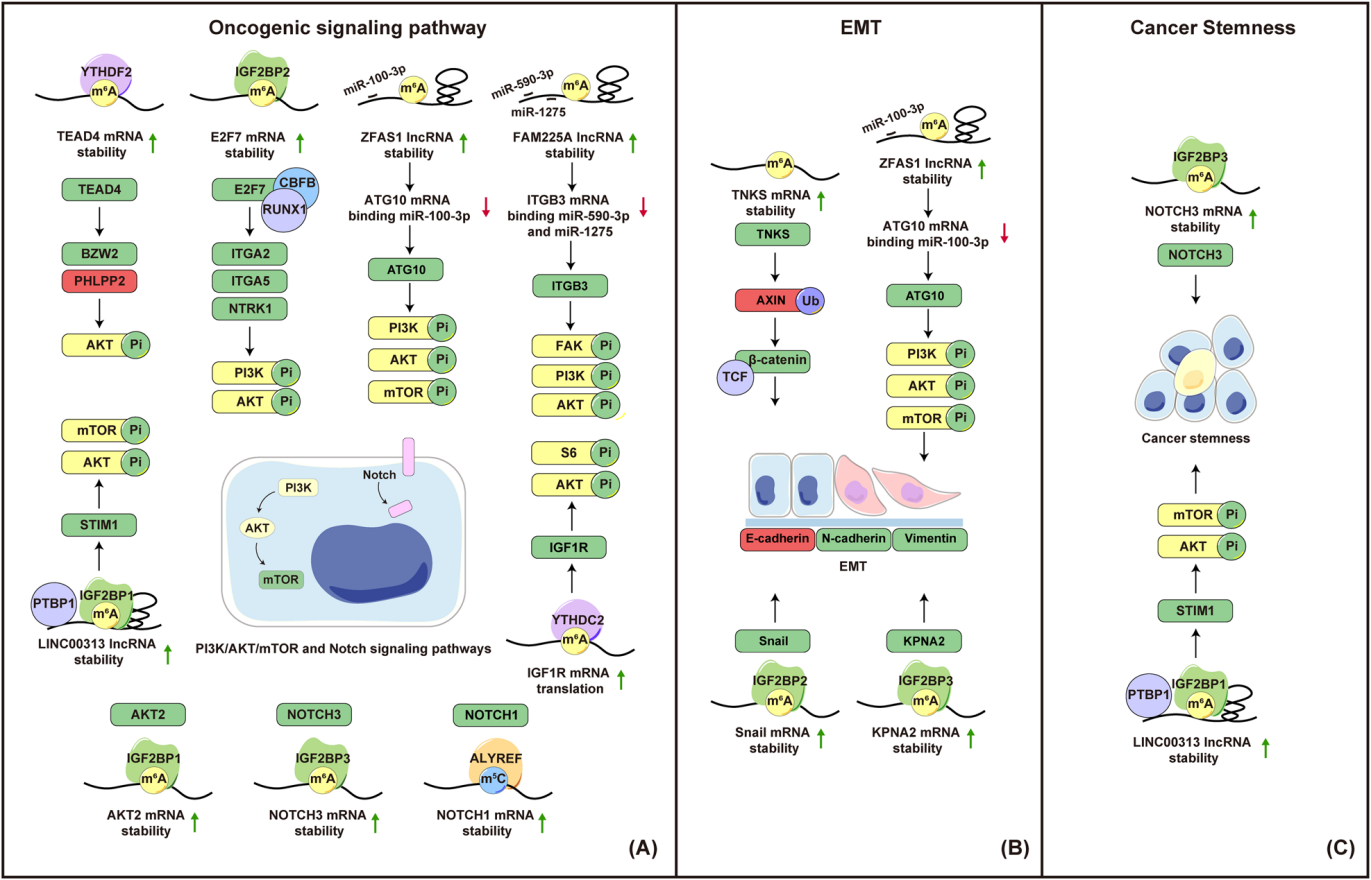
Roles of RNA Modifications in Oncogenic Signaling Pathways, EMT, and Cancer Stemness in NPC. **A Oncogenic Signaling Pathways:** (1) m^6^A modification of TEAD4 mRNA, recognized by YTHDF2, stabilizes TEAD4, leading to its upregulation, which promotes BZW2 transcription and inhibits PHLPP2, thus activating the AKT pathway. (2) m^6^A-modified E2F7 mRNA is stabilized by IGF2BP2, increasing E2F7 expression; E2F7, along with RUNX1 and CBFB, transcriptionally activates ITGA2, ITGA5, and NTRK1, activating the PI3K/AKT pathway. (3) The lncRNA ZFAS1, stabilized by m^6^A, acts as a molecular sponge for miR-100-3p, enhancing ATG10 expression and activating the PI3K/AKT pathway. (4) m^6^A-modified lncRNA FAM225A acts as a sponge for miR-590-3p and miR-1275, activating ITGB3 and subsequently the FAK/PI3K/AKT pathway. (5) In Taxol-resistant NPC cells, IGF2BP1 stabilizes m^6^A-modified AKT2 mRNA, increasing AKT2 expression. (6) In radioresistant NPC cells, YTHDC2 stabilizes m^6^A-modified IGF1R, enhancing AKT and S6 phosphorylation. (7) IGF2BP3 stabilizes m^6^A-modified NOTCH3 mRNA, activating Notch pathway. (8) ALYREF, recognizing NSUN2-mediated m^5^C-modified NOTCH1, stabilizes NOTCH1 mRNA, activating Notch signaling. **B EMT:** (1) m^6^A modification stabilizes TNKS mRNA, enhancing its expression, which promotes AXIN degradation via ubiquitination and activates β-catenin/TCF signaling. (2) The lncRNA ZFAS1, stabilized by m^6^A, acts as a molecular sponge for miR-100-3p, activating the PI3K/AKT pathway and promoting EMT. (3) m^6^A-modified Snail1 mRNA, recognized by IGF2BP2, is stabilized, increasing Snail1 expression. (4) KPNA2 mRNA, stabilized by IGF2BP3, is upregulated, promoting EMT. **C Cancer Stemness:** (1) IGF2BP3 stabilizes NOTCH3 mRNA in an m^6^A-dependent manner, activating the Notch pathway and promoting cancer stemness. (2) LINC00313, stabilized by m^6^A, recruits PTBP1, enhancing the STIM1/AKT axis and promoting cancer stemness. Green squares or upward arrows represent upregulated targets, while red squares or downward arrows indicate downregulated targets.

The PI3K/AKT/mTOR signaling pathway is critical for fundamental cellular functions, including growth, proliferation, and metabolism. This pathway is often abnormally activated in aggressive tumors, notably in NPC [144]. In NPC, m^6^A methylation facilitated by WTAP and recognized by YTHDF2 significantly stabilizes TEAD4, leading to its increased levels. This elevated TEAD4 enhances NPC cell migration, invasion, metastasis, and resistance to cisplatin by transcriptionally upregulating BZW2 and suppressing the phosphatase PHLPP2, thereby activating the oncogenic AKT pathway [103] (Fig. 3A and Table 1). E2F7, stabilized by VIRMA-mediated m^6^A modification and IGF2BP2, in cooperation with RUNX1 and CBFB, transcriptionally activates ITGA2, ITGA5, and NTRK1, leading to activation of PI3K/AKT pathway and promoting NPC progression [74] (Fig. 3A and Table 1). The lncRNA ZFAS1, stabilized and expressed due to m^6^A modification by METTL3, functions as a molecular sponge for miR-100-3p, which in turn activates the PI3K-AKT pathway, promoting proliferation, epithelial-mesenchymal transition (EMT), and autophagy [63] (Fig. 3A and Table 1). Similarly, the lncRNA FAM225A, also m^6^A-modified by METTL3, sponges miR-590-3p and miR-1275, thereby promoting ITGB3 then activating the FAK/PI3K/AKT pathway, thus facilitating proliferation, migration, invasion, and metastasis [145] (Fig. 3A and Table 1). Furthermore, IGF2BP3 facilitates EMT, promoting NPC cell migration and invasion through AKT/mTOR signaling [88] (Fig. 3A and Table 1). In Taxol-resistant NPC cell lines, IGF2BP1 increases AKT2 expression by stabilizing its mRNA via m^6^A recognition [109] (Fig. 3A and Table 1). In the context of radioresistance, YTHDC2 promotes AKT and S6 phosphorylation by recognizing and stabilizing IGF1R m^6^A modifications [86] (Fig. 3A and Table 1).

The Notch signaling pathway is widely overactivated in multiple cancers, regulating stemness and metastasis. In NPC, inhibiting Notch signaling reduces proliferation and increases sensitivity to treatment. RNA modification is likely important in this regulation. Specifically, IGF2BP3 stabilizes NOTCH3 mRNA in an m^6^A-dependent manner, ensuring the activation of the NOTCH3 pathway. This activation enhances the survival and tumor-initiating capabilities of disseminated tumor cells, leading to significant tumor metastasis in NPC [85] (Fig. 3A and Table 1). The m^5^C reader ALYREF recognizes NSUN2-mediated NOTCH1 m^5^C modification, enhancing its RNA stability, thereby promoting activation of Notch signaling and facilitating NPC metastasis [79] (Fig. 3A and Table 1).

### Regulation of EMT and cancer stemness

EMT is a cellular process where epithelial cells transform into a mesenchymal state, allowing for increased mobility. This transformation is crucial for various developmental processes, including gastrulation, neural crest formation, heart valve formation, and wound healing [146]. In pathological conditions, EMT contributes to organ fibrosis, cancer progression, and metastasis. Core transcription factors such as Snail1/2, Twist1/2, and Zeb1/2 drive EMT by inhibiting epithelial markers like E-cadherin and ZO-1, and promoting mesenchymal markers like N-cadherin, vimentin, and fibronectin [147]. These alterations allow carcinoma cells to lose their epithelial polarity and junctions, adopt mesenchymal traits, and gain enhanced migratory and invasive abilities. Consequently, EMT is essential for cancer metastasis, enabling tumor cells to spread from the primary site to distant organs. RNA modifications significantly impact cancer metastasis, particularly during EMT. Research shows that the m^6^A writer METTL3 is a key regulator in cancers such as liver, gastric, and ovarian cancers, as it promotes EMT and tumor progression. Other m^6^A regulators, like ALKBH5, influence cancer cell behavior by modulating factors and signaling pathways related to EMT [148]. In NPC, METTL3 facilitates EMT by modulating key markers, such as downregulating E-cadherin and upregulating N-cadherin and Snail [64] (Table 1). METTL3 also stabilizes transcripts like Tankyrase, which activates β-catenin/TCF signaling, thereby enhancing migration, invasion, and metastasis [65] (Table 1). Additionally, METTL3 stabilizes lncRNA ZFAS1, which sponges miR-100-3p to activate the PI3K/AKT pathway, encouraging proliferation, autophagy, and EMT [63] (Table 1). The m^6^A reader IGF2BP2 identifies Snail transcripts [64], while IGF2BP3 stabilizes m^6^A-modified KPNA2 transcripts, which increases vimentin expression and decreasing E-cadherin levels, further advancing EMT [87] (Fig. 3B and Table 1).

Cancer stem cells (CSCs) are a small subset of cancer cells that have a high capacity for self-renewal and tumor initiation. CSCs play key roles in tumor initiation, heterogeneity, metastasis, and resistance to therapy [149]. EMT enhances CSC characteristics. Research suggests that EMT is crucial for gaining stem-like traits and influences tumor formation, metastasis, dormancy, and relapse in various cancers, including breast, ovarian, pancreatic, squamous cell carcinoma, colon, prostate, and gastric cancers [146]. Recent studies reveal the significant role of m^6^A modulation in various CSCs. In breast cancer, ALKBH5 affects the number and activity of CSCs, especially under hypoxic conditions [150]. In colorectal cancer, METTL3 influences stem cell frequency and tumor progression by regulating SOX2 mRNA stability [151]. In glioblastoma, studies indicate that METTL3 affects the growth and radiation sensitivity of stem-like cells [105, 152, 153]. In NPC, METTL3 stabilizes LINC00313, which recruits PTBP1, promoting STIM1 expression and enhancing cancer stemness [66] (Fig. 3C and Table 1). IGF2BP3 stabilizes NOTCH3 mRNA in an m^6^A-dependent manner, activating the Notch signaling pathway. This activation enhances the survival and tumor-initiating capabilities of disseminated tumor cells, leading to significant tumor metastasis [85] (Fig. 3C and Table 1). Overall, these findings underscore the critical role of m^6^A modifications and related signaling pathways in regulating EMT and cancer stemness in NPC, offering potential therapeutic targets.

### Regulation of apoptosis and ferroptosis

**Fig. 4:**
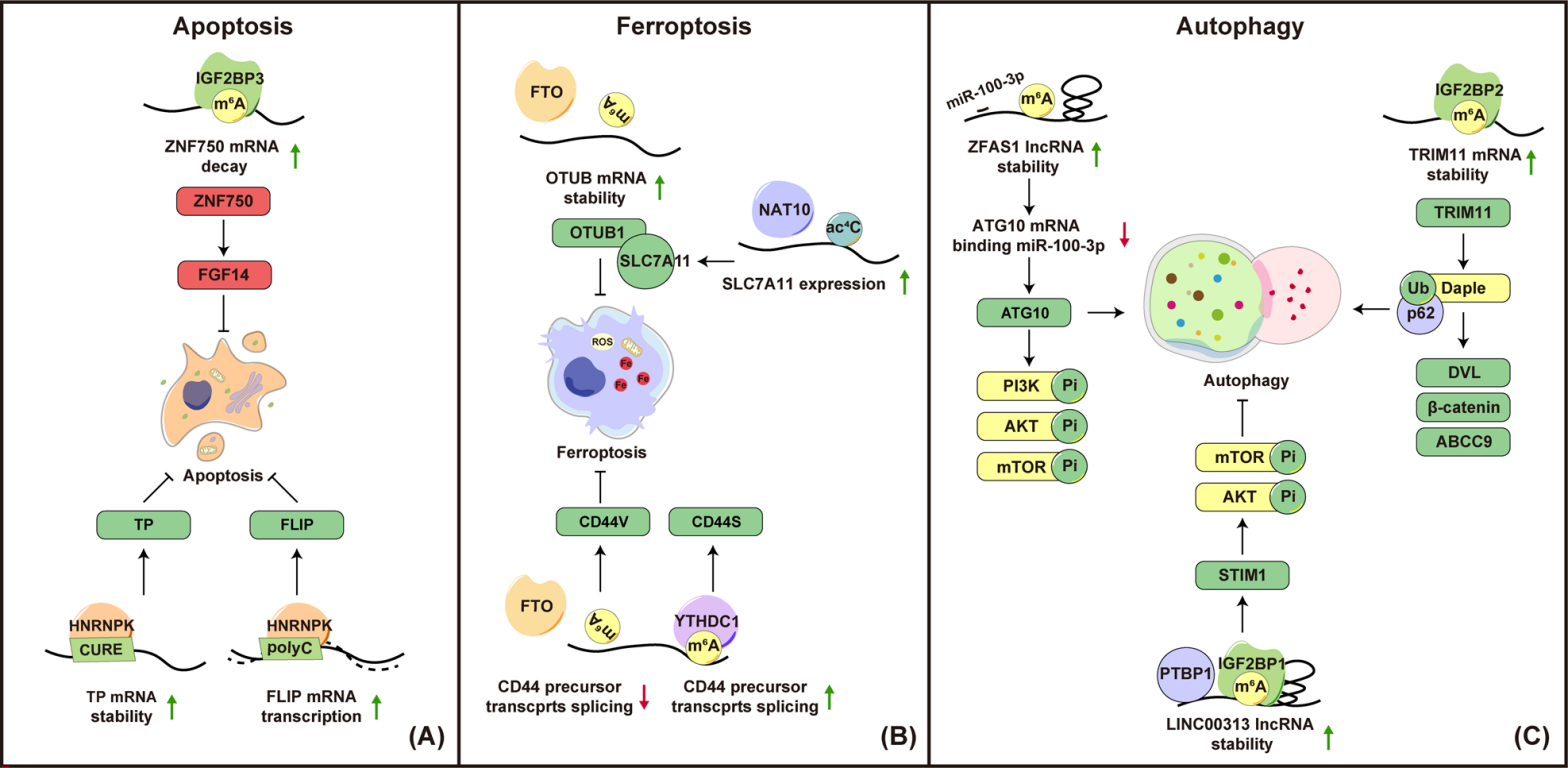
Roles of RNA Modifications in Apoptosis, Ferroptosis, and Autophagy in NPC. **A Apoptosis:** (1) m^6^A modification of ZNF750, recognized by IGF2BP3, facilitates ZNF750 degradation, disrupting the FGF14 axis, inhibiting apoptosis, and supporting NPC growth. (2) HNRNPK suppresses apoptosis in NPC cells by activating FLIP transcriptionally. (3) In the cytoplasm, HNRNPK stabilizes TP, blocking apoptosis in tumor cells. **B Ferroptosis:** (1) In radioresistant NPC cells, FTO upregulation removes m^6^A from OTUB1 transcripts, stabilizing OTUB1 and allowing it to recruit SLC7A11, which inhibits radiation-induced ferroptosis, promoting tumorigenesis. (2) NAT10 enhances sorafenib resistance by upregulating SLC7A11 expression through ac4C acetylation. (3) FTO-mediated demethylation of CD44 transcripts reduces YTHDC1-mediated splicing, increasing CD44V levels, which inhibits irradiation-induced ferroptosis and contributes to radioresistance. **C Autophagy:** (1) ZFAS1, acting as a sponge for miR-100-3p, increases ATG10 expression, driving autophagy and supporting cancer progression. (2) LINC00313, stabilized by m^6^A recognition via IGF2BP1, interacts with PTBP1 to upregulate STIM1, activating the AKT/mTOR pathway, which suppresses autophagy and enhances cancer stemness. (3) m^6^A-modified TRIM11, stabilized by IGF2BP2, upregulates its expression, promoting Daple ubiquitination and p62-mediated selective autophagy. Reduced Daple levels lead to DVL upregulation, activating the β-catenin/ABCC9 axis and contributing to drug resistance. Green squares or upward arrows represent upregulated targets, while red squares or downward arrows indicate downregulated targets.

Under normal conditions, the organism maintains a balance between cell proliferation and death, eliminating aging, dysfunctional, or mutated cells through programmed cell death [154]. Insufficient cell death can lead to diseases characterized by excessive proliferation, such as cancer. Apoptosis, the primary form of programmed cell death, involves two main pathways: the mitochondrial (intrinsic) pathway and the death receptor (extrinsic) pathway. The mitochondrial pathway is triggered by cellular stress or developmental signals that alter Bcl-2 family proteins, which regulate mitochondrial membrane integrity. Key proteins like Bax and Bak cause mitochondrial outer membrane permeabilization (MOMP), releasing intermembrane space proteins into the cytosol. This release activates caspases, enzymes that cleave specific cellular substrates, resulting in the characteristic features of apoptosis, including chromatin condensation and membrane blebbing. The death receptor pathway starts when specific ligands bind to TNF receptors on the cell surface, leading to the recruitment and activation of caspase-8. Caspase-8 can also promote MOMP, linking the extrinsic and intrinsic pathways. Both pathways converge on the activation of executioner caspases, driving the morphological and biochemical changes characteristic of apoptosis [155]. RNA modifications can influence tumorigenesis through various mechanisms, including their effects on apoptosis [156, 157]. For instance, proteins like METTL3 can promote or inhibit apoptosis by modulating the translation or degradation of Bcl-2 family proteins [158–160]. YTHDF2-mediated degradation of TNFRSF2 interrupts the TNF signaling pathway, preventing apoptosis in AML cells [161]. In NPC, METTL3-mediated m^6^A modification reduces ZNF750 expression, weakening its tumor-suppressor function in the ZNF750-FGF14 axis, which inhibits apoptosis and promotes NPC growth [130] (Fig. 4A and Table 1). While additional regulatory mechanisms of RNA modifications in NPC apoptosis remain unclear, studies suggest that the m^6^A reader HNRNPK inhibits apoptosis in NPC cells by transcriptionally activating FLIP [94] (Fig. 4A and Table 1). Furthermore, cytoplasmic HNRNPK enhances the stability and expression of thymidine phosphorylase (TP), which has been shown to inhibit tumor cell apoptosis [95]. Although HNRNPK interacts with the CU-rich element of TP mRNA [95], it is also possible that it recognizes m^6^A modifications on TP mRNA (Fig. 4A and Table 1).

Recently, alongside apoptosis, other forms of programmed cell death, including pyroptosis, ferroptosis, cuproptosis, and disulfidptosis, have gained increasing attention, particularly for their impact on tumor progression. Ferroptosis is a type of non-apoptotic cell death caused by the accumulation of toxic, iron-dependent phospholipid peroxides in cell membranes [154, 162]. This process is influenced by metabolic enzymes like ASCL4 and LPCAT3, which generate oxidizable membrane lipids, and iron-dependent enzymes such as arachidonate lipoxygenases, which oxidize these lipids [163]. Additionally, ferroptosis can be induced by the suppression of system Xc − , a cystine/glutamate antiporter composed of two major subunits, SLC7A11 (solute carrier 7A11) and SLC3A2 (solute carrier 3A2). The inhibition of ferroptosis in tumors is closely associated with tumor development, and RNA modifications play a significant role in this process. In prostate cancer, the upregulation of YTHDF1 promotes PD-L1 expression in an m^6^A-dependent manner, reducing T-cell toxicity and inhibiting ferroptosis, which leads to poor clinical outcomes [164]. Similarly, in hepatoblastoma, SLC7A11 upregulation, driven by IGF2BP1 through METTL3-m^6^A modification, supports tumor proliferation and inhibits ferroptosis [165]. In NPC, recent studies have observed that radiation or chemotherapy increases lipid peroxidation, thereby inducing ferroptosis [166–168]. Radioresistant NPC cells, which often exhibit suppressed ferroptosis, become highly sensitive to radiation when treated with erastin, a small molecule that targets SLC7A11, leading to glutathione depletion and ferroptosis [81]. Notably, in these cells, FTO is significantly upregulated, which removes the m^6^A modification from the OTUB1 transcript. OTUB1, a deubiquitinase that stabilizes SLC7A11, is consequently upregulated, inhibiting radiation-induced ferroptosis and promoting tumorigenesis [81] (Fig. 4B and Table 1). Similarly, NAT10 contributes to sorafenib resistance, cell proliferation, and metastasis by enhancing SLC7A11 expression through ac4C acetylation [169] (Fig. 4B and Table 1). Furthermore, upregulation of lncRNA HOTAIRM1 enhances resistance to ferroptosis and irradiation by modulating the FTO-YTHDC1-CD44 axis. HOTAIRM1 interacts with the FTO protein, inducing m^6^A demethylation of CD44 precursor transcripts. This demethylation prevents their recognition by YTHDC1 and subsequent alternative splicing, leading to increased levels of CD44V. Elevated CD44V suppresses irradiation-induced ferroptosis, thereby contributing to radioresistance [82] (Fig. 4B and Table 1). While current studies are still unraveling the complex regulatory mechanisms of RNA modifications in various forms of cell death in NPC, necessitating further research to fully elucidate these processes.

### Regulation of autophagy

Autophagy is a crucial homeostatic process that degrades and recycles cellular materials. It is orchestrated by a series of autophagy-related (ATG) gene products, which facilitate the formation of autophagosomes, double-membrane vesicles that engulf cellular damaged or dysfunctional proteins and organelles. These autophagosomes then fuse with lysosomes, where their contents are degraded by lysosomal hydrolases, with the resulting products recycled to support cellular function [170]. In cancer, autophagy plays a dual role, serving both tumor-suppressive and tumor-promoting functions depending on the disease stage and genetic context [171]. Recent research has revealed that autophagy is intricately regulated by epigenetic modifications, including those on DNA, RNA, and proteins. Notably, RNA modifications, especially m^6^A, have gained considerable attention in this regard. During starvation-induced autophagy, the m^6^A reader YTHDF3 is significantly upregulated. The m^6^A writer METTL3 facilitates the hypermethylation of FOXO3 mRNA, a modification that YTHDF3 recognizes, thereby enhancing the translation of FOXO3 and promoting the autophagy process [172, 173]. In non-small cell lung cancer (NSCLC) and small cell lung cancer (SCLC), METTL3-mediated m^6^A modification enhances autophagy and mitophagy, contributing to gefitinib resistance and chemoresistance, respectively [174, 175]. In hepatocellular carcinoma (HCC), elevated m^6^A modification of ATG5, driven by WTAP and recognized by YTHDC2, induces autophagy and promotes ferroptosis, thereby inhibiting HCC progression [176]. Under hypoxic conditions, the m^6^A reader protein YTHDF1 drives autophagy and cancer progression by enhancing the translation of ATG2A and ATG14 [177]. In contrast, depletion of METTL3-mediated m^6^A modification of FOXO3 triggers autophagy and increases resistance to sorafenib [178]. In esophageal cancer, m^7^G modification of tRNA has also been found to influence autophagy [179]. These studies underscore the importance of RNA modifications in autophagy regulation.

In NPC, recent studies also have highlighted the significance of RNA modifications in autophagy regulation. Specifically, long non-coding RNAs ZFAS1 and LINC00313 are m^6^A-modified by METTL3, with LINC00313 being stabilized by IGF2BP1. Both RNAs are significantly upregulated in NPC and are associated with poor clinical outcomes. Mechanistically, ZFAS1 acts as a molecular sponge for miR-100-3p, inhibiting its activity and thus increasing the expression of the autophagy-related gene ATG10, which promotes autophagy and cancer progression [63] (Fig. 4C and Table 1). On the contrary, LINC00313 interacts with PTBP1 to upregulate STIM1, activating the AKT/mTOR pathway, which suppresses autophagy and enhances stemness in NPC cells [66] (Fig. 4C and Table 1). Additionally, METTL3-mediated m^6^A modification of TRIM11, recognized by IGF2BP2, enhances mRNA stability, leading to its upregulation. This upregulation is associated with decreased overall survival and progression-free survival in NPC patients. TRIM11 mediates Daple ubiquitination, promoting its degradation through p62-selective autophagy. Reduced Daple levels lead to DVL upregulation, activating the β-catenin/ABCC9 axis and contributing to multidrug resistance [180] (Fig. 4C and Table 1).

### Regulation of metabolism

Cellular metabolism is a dynamic network that enables cells to meet their energy and biosynthetic demands. In malignant cells, metabolic reprogramming plays a key role in tumorigenesis, progression, metastasis, and chemoresistance. Dysregulated RNA modifiers significantly contribute to these metabolic changes by targeting metabolic enzymes, transporters, and related transcription factors or pathways, thereby impacting glucose, lipid, amino acid, and mitochondrial metabolism [181]. Glucose metabolism is essential for tumor cells, with the Warburg effect being a hallmark where tumor cells prefer glycolysis over oxidative phosphorylation (OXPHOS) even in oxygen-rich environments [182]. m^6^A RNA methylation plays a crucial role in this process. For example, METTL3 induces m^6^A modifications on GLUT1 mRNA, enhancing glucose uptake and lactate production, which in turn activates mTORC1 signaling and promotes colorectal cancer progression [183]. In papillary thyroid cancer, low expression of FTO upregulates m^6^A methylation of apolipoprotein E (APOE) mRNA, modulating the IL-6/JAK2/STAT3 pathway, thus promoting tumor glycolysis and growth [184]. YTHDC1 mitigates glycolysis by counteracting the Warburg effect, which hinders pancreatic cancer development [185]. RNA modification also plays a significant role in lipid metabolism. FTO promotes lipid droplet formation in esophageal cancer cells through HSD17B11 gene expression, via a YTHDF1-dependent mechanism [186]. Knockdown of METTL5-mediated 18S rRNA m^6^A reduces triglycerides, cholesterol, and free fatty acids, thereby inhibiting HCC progression [187].

**Fig. 5:**
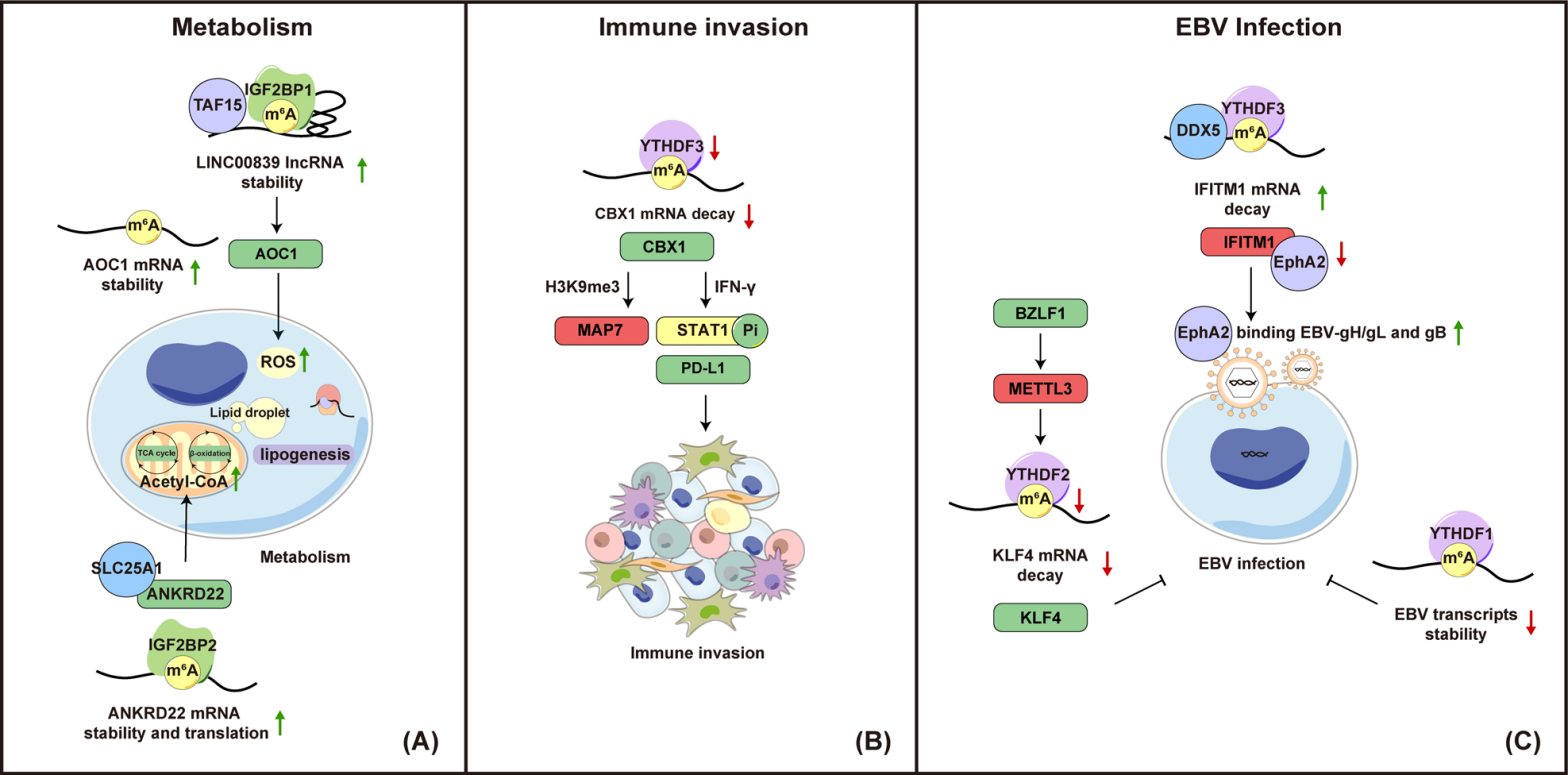
Roles of RNA modifications in metabolism, immune invasion, and EBV infection in NPC. **A Metabolism:** (1) METTL14-mediated m^6^A modifications on ANKRD22 mRNA, recognized by IGF2BP2, stabilize ANKRD22, enhancing its translation. ANKRD22 interacts with the citrate transporter SLC25A1, increasing intracellular acetyl-CoA levels to drive de novo lipid synthesis. (2) METTL14 stabilizes AOC1 mRNA via m^6^A modifications. AOC1 degrades polyamines, producing ROS as a byproduct. (3) LINC00839, stabilized by IGF2BP1, recruits TAF15 to activate AOC1 transcription. **B Immune Invasion:** Reduced levels of the m^6^A reader YTHDF3 in metastatic NPC impair CBX1 decay, resulting in elevated CBX1 levels. CBX1 upregulates PD-L1 via IFN-γ-STAT1 signaling, promoting immune evasion, and concurrently enhances cell proliferation by repressing MAP7 through H3K9me3-modified heterochromatin formation. **C EBV Infection:** (1) YTHDF1 destabilizes m^6^A-modified EBV transcripts, suppressing EBV infection and replication. (2) IFITM1 competes with EBV glycoproteins gH/gL and gB for EphA2 binding. YTHDF3, with DDX5, accelerates IFITM1 mRNA degradation via m^6^A, enhancing EphA2-mediated EBV infection. (3) During EBV infection, the immediate-early protein BZLF1 binds the METTL3 promoter, lowering METTL3 expression. This reduction decreases m^6^A modifications on KLF4 mRNA, preventing YTHDF2-mediated degradation, resulting in elevated KLF4 protein levels and promoting EBV infection in NPE cells. Green squares or upward arrows represent upregulated targets, while red squares or downward arrows indicate downregulated targets.

In NPC, recent research underscores METTL14’s significant role in promoting tumor progression by altering lipid metabolism through m^6^A RNA modifications. METTL14 facilitates m^6^A modifications on ANKRD22 mRNA, a critical factor in lipid metabolism reprogramming. These modifications are recognized by IGF2BP2, stabilizing it and enhancing its translation. This results in increased ANKRD22 expression in NPC tissues. ANKRD22, located in the mitochondria, interacts with the citrate transporter SLC25A1, leading to elevated intracellular acetyl-CoA levels. This rise in acetyl-CoA facilitates de novo lipid synthesis, which is vital for maintaining cellular membranes and energy storage, thereby promoting cancer growth and metastasis [58] (Fig. 5A and Table 1). Beyond its role in lipid metabolism, METTL14 also stabilizes AOC1 mRNA via m^6^A modifications. AOC1, a secreted diamine oxidase, degrades polyamines like putrescine and histamine, producing hydrogen peroxide (ROS) in the process. While excessive ROS can be harmful, moderate levels of ROS can promote tumor progression [188] (Fig. 5A and Table 1). Beyond METTL14, studies show that VIRMA and IGF2BP1 stabilize LINC00839 in an m^6^A-dependent manner. LINC00839 recruits TAF15 to activate AOC1 transcription, thereby enhancing NPC proliferation, migration, and invasion [110] (Fig. 5A and Table 1). These findings indicate a prominent role for RNA modification in the regulation of metabolism in NPC.

### Regulation of immune invasion

The tumor microenvironment (TME) is characterized by an immunosuppressive state that downregulates immune cell functions and facilitates tumor immune evasion. Typically, antitumor responses involve activated CD8 + T cells, which recognize tumor antigens presented by antigen-presenting cells (APCs) and exert cytotoxic effects to destroy tumor cells. However, tumor cells often emit suppressive signals through various mechanisms, including an immunosuppressive TME, reducing HLA-1 expression, and upregulating immune checkpoint proteins like PD-1 and PD-L1 [189]. RNA modification is central to regulating immunosuppressive factors in tumors. For instance, m^6^A methylation significantly influences PD-1/PD-L1 regulation through splicing, stability, and translation, ultimately facilitating immune evasion in various tumors [189, 190]. The m^5^C modification, controlled by factors such as NSUN2, TET2, and YBX1, also affects tumor immune responses by altering immune cell infiltration and the expression of immune checkpoints [78, 191, 192]. A recent study reveals that EBV EBNA1 promotes the degradation of the METTL3 protein via the K48-linked ubiquitin pathway, decreasing TLR9 m^6^A modification and YTHDF1-enhanced translation, thus promoting tumor immune evasion [68] (Table 2). In NPC, ongoing EBV infection often decreases the immunogenicity of cancer cells and modifies the tumor microenvironment, enabling tumor cells to evade immune clearance [193]. Recent clustering analysis of m^6^A-related molecular patterns suggests that elevated expression of specific m^6^A regulators may be associated with increased immunosuppression in NPC [194]. In metastatic NPC, the histone methylation reader CBX1 is upregulated due to reduced levels of the m^6^A reader YTHDF3, which recognizes and degrades its m^6^A-modified transcripts. Elevated CBX1 expression promotes immune evasion by increasing PD-L1 expression through IFN-γ-STAT1 signaling. Concurrently, it also enhances NPC cell proliferation by repressing MAP7 transcription via heterochromatin formation, mediated by CBX1 binding to H3K9me3 [84] (Fig. 5B and Table 1).

### Regulation of EBV infection

EBV infection is closely linked to NPC, where both viral latent and lytic phases play crucial roles. A recent study uncovers distinct m^6^A modifications in EBV transcripts within NPC biopsies, patient-derived xenograft tissues, and cells at various EBV infection stages. YTHDF1 destabilizes identified m^6^A-modified EBV transcripts, leading to m^6^A-dependent suppression of EBV infection and replication. In addition, knocking down METTL3 or METTL14 enhances EBV infection, while ALKBH5 knockdown exhibits an opposing effect [104] (Fig. 5C and Table 2). Another study demonstrates that IFITM1 competes with EBV-gH/gL and gB for binding to EphA2, while YTHDF3 accelerates IFITM1 mRNA degradation through m^6^A modification in collaboration with DDX5, thereby promoting EphA2-mediated EBV infection [195] (Fig. 5C and Table 2). For lytic replication, knocking down METTL3, METTL14, YTHDF2, or YTHDF3 all demonstrate a promoting effect. On the other hand, EBV infection and lytic reactivation suppress YTHDF1 expression, indicating positive feedback to facilitate EBV infection [104] (Table 2). There exists other positive feedback loop between EBV and host molecules through cellular mRNA m^6^A levels, fostering viral infection. For example, during EBV infection, the immediate-early protein BZLF1 interacts with the promoter of METTL3, suppressing its expression. This reduction in METTL3 leads to decreased m^6^A modification on KLF4 mRNA, preventing its degradation by the m^6^A reader YTHDF2. As a result, KLF4 protein levels surge, facilitating EBV’s infection of nasopharyngeal epithelial cells [69] (Fig. 5C and Table 2).

In B lymphoma cells, EBV infection influences the host cellular m^6^A epitranscriptome during the pre-latent phase. The emergent m^6^A modifications are preferentially distributed in the 3’UTR of the transcripts, whereas the lost m^6^A modifications preferentially distributed in the CDS [196] (Table 2). Another study indicates no significant changes in the distribution from latent to lytic reactivation [197] (Table 2). In the pre-latent phase, viral transcripts EBNA2 and BHRF1, but not LMP1, contain m^6^A modifications in their CDS regions. Knocking down METTL3 or YTHDF1 decreases EBNA2, while knocking down FTO, YTHDF2, and YTHDF3 increases EBNA2 [196] (Table 2). In the EBV lytic cycle, METTL3 plays a crucial role in EBV progeny production but has a marginal effect on viral DNA replication. Both early (BZLF1, BHRF1, BMRF1) and late (BKRF4, BALF4) lytic gene transcripts decrease when knocking out METTL3, potentially regulated differentially through stability and translation. Notably, knocking out METTL3 decreases the EBV-positive Akata cell viability during the lytic phase rather than without lytic induction [198] (Table 2). Meanwhile, ALKBH5 dramatically decreases during EBV lytic reactivation, leading to increased IFN production through specific gene transcripts hypermethylated, such as DTX4 and TYK2, thereby reducing the viral replication copies [197] (Table 2). METTL14 also plays a pivotal role in EBV’s infection and subsequent oncogenesis, with levels spiking during EBV latent infection but declining during the lytic phase. Knocking down METTL14 diminishes latent gene expression but enhances lytic gene expression. The critical EBV latent antigen EBNA3C, pivotal for viral-mediated cell transformation, is upregulated through METTL14-mediated m^6^A modification. This sets off a feedback loop that boosts METTL14 transcription and stabilizes its protein [199] (Table 2). YTHDF2 demonstrates a universal role in suppressing EBV gene expression and EBV replication. Depletion of YTHDF2 promotes most EBV immediate early, early, and late genes even without lytic induction, and enhances EBV copy numbers upon lytic induction. Mechanistically, CASP8 transcript is m^6^A-modified and bound by YTHDF2. Depletion of YTHDF2 promotes CASP8 mRNA and caspase-8 protein level, hence activating and cleaving PIAS1 and YTHDF2 itself, favoring EBV lytic replication [200] (Table 2). PIAS1 acts as a SUMOylator for YTHDF2, and together they restrict EBV replication. When YTHDF2 lacks SUMOylation, its binding to EBV transcripts decreases, resulting in increased mRNA stability and enhanced viral replication [201] (Table 2). Additionally, EBV impacts the host’s m^6^A system. EBNA1 facilitates the degradation of the METTL3 protein via the K48-linked ubiquitin pathway, reducing TLR9 m^6^A modification and YTHDF1-enhanced translation, thus promoting tumor immune evasion [68] (Table 2).

In EBV-associated gastric cancer (EBVaGC), there is significant decrease in m^6^A modifications compared to EBV-negative cases. The decrease is attributed to reduced WTAP levels triggered by EBV-encoded small RNA, EBER1, through the NF-κB signaling pathway [202] (Table 2). High expression of FTO is also noted, correlating with a favorable prognosis. EBV induces FTO expression through the transcription factor MYC, causing reduced m^6^A modification of FOS, which is recognized and stabilized by IGF2BP1/2 through m^6^A [203] (Table 2). These mechanisms contribute to restraining the progression and metastasis of EBVaGC. However, METTL3 upregulation is also reported correlating with poor OS and metastasis. An EBV-generated circular RNA, circRPMS1, recruiting Sam68 to transactivate METTL3 and induce its expression, contributing to EBVaGC progression [204] (Table 2).

## Conclusions

The epitranscriptomic machinery plays a crucial role in regulating various biological processes and cancer development. Global RNA modification levels may be unreliable as biomarkers for tumor progression or prognosis, as RNA modifications at different genes or loci can lead to distinct molecular outcomes, either promoting or suppressing tumorigenesis. Furthermore, different RNA modification regulators have specific target sites, and their expression can vary across different tumor samples. Nevertheless, in specific cellular contexts, changes in RNA modifications at particular sites can produce clear oncogenic or tumor-suppressive effects. These molecular alterations can serve as indicators of tumor progression and potential therapeutic targets. In NPC, the overall upregulation of m^6^A modifications in mRNAs, lncRNAs, and rRNAs compared to NPE indicates a significant role in NPC development. Additionally, m^6^A regulators, particularly the METTL3-METTL14 methyltransferase complex and the rRNA m^6^A writer METTL5/TRMT112, are consistently upregulated across multiple studies. Similarly, m^5^C writers such as NOP2, NSUN2, and the tRNA m^7^G writer METTL1/WDR4 are significantly upregulated in NPC, highlighting widespread changes in the epitranscriptomic landscape and supporting the idea that RNA hypermethylation plays a key role in NPC pathogenesis. To date, the functions and clinical significance of other RNA modifications (e.g., m^1^A, m^6^Am, Nm, ac^4^C, and pseudouridine), along with their respective writers, erasers, and readers, remain unclear in NPC. These unresolved areas warrant further investigation.

RNA modifications regulate gene expression by influencing RNA stability, translation, processing, and export. Early studies have shown that m^6^A-modified RNA typically exhibits lower stability, resulting in decreased abundance. YTHDF2 facilitates the degradation of m^6^A-marked RNA by recruiting the CCR4-NOT complex and working in concert with other proteins to achieve this outcome. However, in specific cellular contexts, YTHDF2 stabilizes transcripts, displaying an opposite effect. Similarly, other m^6^A reader proteins, such as YTHDF1, YTHDF3, YTHDC proteins, and IGF2BPs, orchestrate the degradation or stability of m^6^A-modified RNA, or enhance its translation by recruiting various cofactors or forming specialized complexes. Additionally, readers such as YTHDC1 and HNRNPA2B1 can regulate gene expression by recruiting splicing factors, facilitating RNA splicing, miRNA processing and maturation, and the export of RNA from the nucleus. Besides post-transcriptional roles, RNA modifications also play a crucial part during transcription. The expression of several transcription factors, such as Snail and E2F7, is regulated by m^6^A modifications. Moreover, m^6^A modifications directly participate in the transcription process. For instance, during RNA synthesis, m^6^A modifications can recruit YTHDC1 to facilitate the release and recycling of RNA polymerase II. m^6^A modifications on enhancer RNA recruit YTHDC1 to form condensates that promote transcription. Furthermore, m^6^A modifications in the nascent RNA promoter and enhancer regions can prevent the repression of RNA-binding integrative complexes. There is also a close relationship between m^6^A modifications and histone modifications. m^6^A modifications can regulate the abundance of transcripts encoding histone modification enzymes, and m^6^A readers can recruit histone modification regulators to alter histone modification levels. Conversely, histone modifications can recruit m^6^A regulators or influence their expression. Additionally, RNA modifications play a significant role in maintaining chromatin stability and promoting DNA repair processes. Despite significant advances in understanding the molecular mechanisms of RNA modifications, ongoing research continues to reveal increasing complexity in these regulatory mechanisms, with many unresolved aspects requiring further exploration. Particularly in the context of malignant tumors such as NPC, the roles and potential clinical significance of these molecular mechanisms remain to be thoroughly investigated.

How do RNA modifications fine-tune the complex cellular processes underlying tumor development and progression? In NPC, RNA modifications regulate key oncogenic pathways, EMT, cancer stem cell properties, programmed cell death, autophagy, metabolism, and immune evasion. Among these modifications, m^6^A methylation plays a particularly critical role. m^6^A modification stabilizes transcripts of oncogenes such as TEAD4 and E2F7, activating signaling pathways like PI3K/AKT/mTOR and Notch, which drive cell proliferation, invasion, metastasis, and drug resistance. m^6^A also influences the expression of EMT-related transcription factors such as Snail, N-cadherin, and E-cadherin, enhancing NPC’s metastatic potential. Additionally, m^6^A plays a crucial role in maintaining cancer stem-like traits. Regarding programmed cell death, m^6^A modifications regulate apoptosis and ferroptosis by modulating the expression of key genes, such as SLC7A11. Furthermore, m^6^A affects autophagy and metabolic reprogramming in NPC, enhancing lipid biosynthesis and ROS production. m^6^A is also instrumental in shaping the TME, contributing to immune evasion. EBV infection, strongly linked to NPC, is also associated with m^6^A modifications. In conclusion, the diverse biological roles of m^6^A in NPC highlight the critical importance of RNA modifications in NPC progression, underscoring their potential as novel therapeutic targets. Looking ahead, many of the mechanisms through which RNA modifications function in biological processes remain unknown. Further deciphering and understanding of these mechanisms could pave the way for innovative therapeutic approaches and improved treatment strategies.
